# Indole primes plant defense against necrotrophic fungal pathogen infection

**DOI:** 10.1371/journal.pone.0207607

**Published:** 2018-11-16

**Authors:** Qinqin Shen, Lijun Liu, Liping Wang, Qiang Wang

**Affiliations:** Institute of Ecological Agriculture, Sichuan Agricultural University, Chengdu, China; Fujian Agriculture and Forestry University, CHINA

## Abstract

Indole is a volatile compound and emitted from plants challenged by insect infestation or mechanic wounding. It has been shown to prime defense against herbivory. Here we identified that indole induced defense either directly or as a priming agent against necrotrophic pathogens *Fusarium graminearum* and *F*. *moniliforme* in maize and *Magnaporthe oryzae* in rice. With indole pretreatment, smaller lesions were developed in infected leaves, as well as less fungal growth. Indole induced H_2_O_2_ burst in the priming stage like other priming substances did. Such priming relied on mitogen-activated protein kinase (MAPK) cascade, which potentially activated downstream defense signaling. In addition, indole priming resulted in earlier and stronger defensive gene expression upon pathogen infection, including genes of jasmonate and phytoalexin biosynthesis, pathogenesis-related proteins (PRs) and anti-oxidant enzymes, which enhanced plant resistance. Meanwhile, H_2_O_2_ was also identified as the priming agent to induce plant defense. Taken together, indole exhibited priming function not only against herbivory but also necrotrophic pathogens. The common emission of indole in plants suggests that it plays important roles as the universal and endogenous priming substance in plant defense.

## Introduction

Plant activates several layers of defense systems in response to pathogen infection, including defensive phytohormones, reactive oxygen species (ROS), specialized metabolites like phytoalexins, physical barriers like callose and lignin, and defense related gene expression. All these defense responses fall into two categories, systemic acquired resistance (SAR) and induced systemic resistance (ISR) [1]. SAR is mediated by salicylic acid (SA) to respond to biotrophic pathogen infection. ISR involves jasmonic acid (JA) and ethylene and defends necrotrophic pathogen invasion.

Either biotrophic or necrotrophic pathogen infection on plants results in failure of reduction-oxidation (Redox) equilibrium and ROS accumulation [2]. ROS play different roles in defense against biotrophic or necrotrophic pathogen. In SAR, ROS are accumulated at infecting sites rapidly upon biotrophic pathogen infection to kill local plant cells and prevent further expansion of pathogens, which is also called hypersensitive reaction (HR). In contrast, necrotrophic fungi stimulate plant ROS accumulation to promote plant cell death, which is beneficial to fungal infection and growth. In such case, ROS scavenging system is activated to eliminate ROS at infection sites to prevent necrotrophic pathogen infection.

ROS are also signal molecules as the secondary messengers in response to environmental elicitation [3,4]. ROS activate a number of defensive signaling pathways, including callose accumulation to strengthen cell wall, antioxidant enzyme upregulation, mitogen-activated protein kinase (MAPK) cascade activation, defensive transcription factor and phytoalexin related gene expression. Among these, MAPK cascade is pivotal through phosphorylation of downstream proteins to promote defense mechanisms such as PR gene expression, ROS scavenging or phytoalexin biosynthesis [5].

Priming is an immune adaptive response and leads to faster and stronger defense response upon herbivory or pathogen infection [6–8]. It needs pretreatment on plants by elicitors to position plants at the alarmed state but not to activate defense reaction directly without attack [9]. Many elicitors have been identified to prime plant resistance, including endogenous phytohormones SA, JA and abscisic acid (ABA), exogenous natural compounds and synthetic elicitors [6–8,10–14]. Although priming mechanism is elusive, two models have been proposed [1]. One of them is accumulation of dormant MAPK proteins by priming for later signaling cascades, another one is explained by epigenetics, i.e. histone modification or DNA methylation for faster expression of defense genes or suppression of defense inhibitors, respectively.

Priming can also be triggered by volatiles [15]. Indole is an endogenous volatile compound and synthesized by indole-3-glycerol phosphate lyase with indole-3-glycerol as the substrate [16,17]. Indole-3-glycerol is also the precursor of tryptophan and anti-herbivore metabolites benzoxazinoids [18]. Indole is detected to be emitted from rice, maize or cotton upon chewing insect infestation or continuous mechanical damage [19–21]. The priming function of indole is reported in maize against herbivory [22]. Indole exposure elicited volatile terpene emission and JA and ABA production in intact plants and resulted in enhanced resistance against incoming insect attack. JA and ABA also play roles in pathogen infection, which arouses the interest to explore the potential priming of indole against necrotrophic fungi.

Here we identified the priming function of indole against necrotrophic fungi in plants. Indole pretreatment induced earlier and stronger expression of PR proteins, phytoalexin biosynthetic genes and anti-oxidant enzyme encoding genes. Such induction was dependent on MAPK cascade and ROS burst at pretreatment stage. Indole priming was observed in maize and rice against different necrotrophic fungi, implicating indole as the universal and endogenous priming substance in plant defense.

## Materials and methods

### Plants and fungi

Maize (*Zea mays*) inbred line Mo17 was grown at 28°C with 16 h light and 8 h dark photoperiod for two weeks. Rice plants (*Oryza sativa* ssp. LTH) were cultivated under 12 h (28°C) light/12 h dark (26°C) for four weeks. *Magnaporthe oryzae* isolate GUY11 was grown on the complete medium for spore production at 25°C under 12 h light/12 h dark cycle. *Fusarium graminearum* and *Fusarium moniliforme* were grown on the spore-production medium to produce spores as described previously [23]. Fungal spores were washed and collected with sterile water and adjusted to the corresponding concentration for leaf inoculation. All plants and fungi were acquired from Sichuan Agricultural University. All experiments were conducted at Chengdu campus of Sichuan Agricultural University (Chengdu, China).

### Indole or H_2_O_2_ priming for detached leaves

Indole (Sigma, CAS: 120-72-9) was dissolved in methanol with the stock concentration of 100 mg mL^-1^. Plant leaves were cut to pieces with 5 cm length and kept in the green-keeping solution (1 mg L^-1^ 6-BA in sterile water, pH 7.0). To test the dose effect, indole with different concentrations (0.1, 0.5, 2.5, 5, 25, 50, 100 mg L^-1^) were added into the green-keeping solution to pretreat leaves for 48 h. The concentration with the best priming effect (50 mg L^-1^) was chosen in the following priming assay. Equal volume of methanol was added as the control. For H_2_O_2_ pretreatment, 10 μM H_2_O_2_ was added into the green-keeping solution to prime defense for 24 h and no H_2_O_2_ was added in the control group. After priming, the leaves were transferred to fresh green-keeping solution without indole or H_2_O_2_, and subsequently infected with fungus spores by punch inoculation as described below. All experiments were performed with three biological replicates.

### Spore punch inoculation

Spores of *F*. *graminearum* and *F*. *moniliforme* were adjusted to the concentration of 5×10^6^ mL^-1^ for maize leaf inoculation. Each leaf was poked to make three equally distributed wounding dots with the needle and 5 μL spores were added on each wound. The inoculated leaves were kept in dark for 12 h and transferred back to 16 h light/8 h dark photoperiod at 26°C for 60 h. The pathogen infection was monitored and infection area was calculated by Image J. The infection percentage was recorded using lesion area divided by whole leaf area. All treatments were repeated with 15 leaves for average infection percentage calculation. *M*. *oryzae* inoculation on rice leaf was performed as the same procedure except the spore concentration of 5×10^5^ mL^-1^. All experiments were performed with three biological replicates.

### Inhibitor treatment

MAPK kinase inhibitor U0126 and proteasome inhibitor MG132 (Selleckchem) were dissolved in DMSO with the stock concentration of 50 mM. After indole priming, leaves were inoculated with spores and inhibitors were added into the green-keeping solution with the final concentration of 100 μM (U0126) or 50 μM (MG132), respectively. Equal amount of DMSO was added as the control. The similar results were obtained from four biological replicated experiments.

### Gene expression analysis

Detached leaves pretreated with indole as above were collected after spore inoculation at 0, 3, 6, 12, 24 an 48 h for RNA extraction with TRNzol reagent (Tiangen, Beijing). cDNA was synthesized with M-MLV reverse transcriptase (Takara) following the manufacture instruction. qRT-PCR was performed on the ABI StepOnePlus with the SYBR Green mix (Gangchi Bio, Hangzhou, China). *Ef1α* was used as the endogenous control gene. All experiments were conducted with three biological replicates. All primers are listed in S1 Table.

### ROS monitoring by staining

H_2_O_2_ accumulation in leaves was recorded by 3, 3'-Diaminobenzidine (DAB) staining. Superoxide radical (O_2_^-^) was stained by Nitro blue tetrazolium chloride (NBT). Leaves were stained with 1 mg mL^-1^ DAB or 0.5 mg mL^-1^ NBT solution at room temperature for 10 h and destained with the destaining solution (ethanol: acetic acid = 3:1, v:v), ready for imaging. Relative amount of H_2_O_2_ was calculated based on pixels taken with Photoshop with the formula as followed: Relative amount of H_2_O_2_ = pixel of H_2_O_2_ area per leaf/ whole pixel of per leaf [24]. H_2_O_2_ in lesions and healthy leaf regions was measured separately. All treatments were repeated with 15 leaves for relative amount of H_2_O_2_ measurement. All experiments were performed with three biological replicates.

### Fungal growth analysis

Fungal growth in leaves was analyzed by qRT-PCR with gDNA as the template. gDNA was extracted from the infected leaves by the CTAB method. qRT-PCR was performed as above. The relative fungal growth was calculated from cycle threshold (Ct) values with the method of 2^-ΔΔCt^. Specifically, *F*. *graminearum* growth was measured as fungal *EF1α* normalized to maize *EF1α* as described previously [25]. *M*. *oryzae Pot2* was used to analyze *M*. *oryzae* growth with rice *Ubiquitin* as the control gene [26]. Three biological replicated experiments were carried out for significance analysis.

### Toxicity of indole to fungi

Toxicity of Indole to fungi was analyzed by adding of 50 mg L^-1^ indole into PDA medium, and pathogen fungi were grown on these media. Methanol was added as the control. After 3–7 d, mycelia in the control reached the edges of plates and diameters of mycelia were measured and compared. All experiments were repeated with three times and significant difference was analyzed.

### Statistics analysis

Significant difference was determined by Tukey’s HSD test or student’s *t*-test based on three or four biological replicates for all experiments.

## Results

### Indole directly induced maize resistance against *F*. *graminearum*

**Fig 1:**
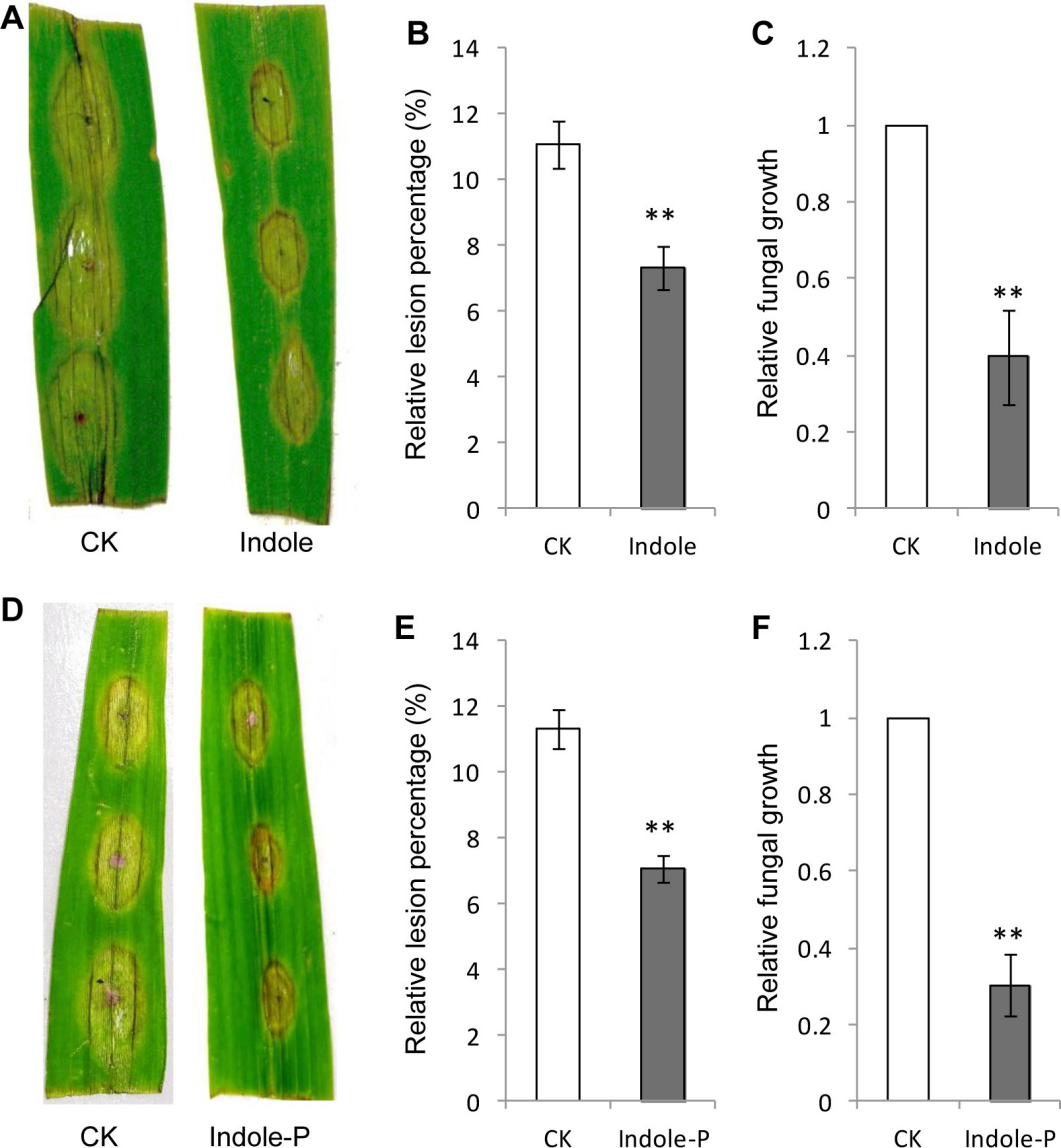
Indole induced resistance against *Fusarium graminearum* infection in detached maize leaves. Direct application (Indole) or pretreatment (Indole-P) of indole resulted in smaller lesions (**A**, **B**, **D**, **E**) and lower relative fungal growth (**C**, **F**). None-treated leaves were used as the control (CK). Relative lesion percentage was calculated with the lesion area divided by leaf area and 15 leaves were used for mean value calculation. The relative fungal growth was analyzed by qRT-PCR with measuring fungal *Ef1α* DNA normalized to maize *Ef1α*. ** indicates significant difference (*P*<0.01, student’s *t*-test). Error bars indicate SE (*n* = 3).

Indole was reported to prime herbivory resistance through inducing JA, ABA biosynthesis and volatile terpene emission [22]. To explore the potential induction in pathogen defense, 50 mg L^-1^ indole was added into the green-keeping solution (1 mg L^-1^ 6-BA in sterile water, pH 7.0) where detached leaves were kept and simultaneously inoculated with spores of the necrotrophic fungus *F*. *graminearum*. After three days, smaller lesions were observed on leaves treated with indole (Fig 1A). The relative fungal growth was also reduced in indole-treated leaves than the control (Fig 1B and 1C). Indole seemed to enhance resistance directly against *F*. *graminearum* infection in maize leaves. Potential toxicity of indole was also tested against pathogen fungi and no significant inhibitory effect was observed for fungal growth on PDA medium (S1 Fig).

### Indole primed maize resistance against pathogen infection

To further explore the priming ability of indole, we pretreated detached maize leaves with indole for 2 d and subsequently inoculated these leaves with *F*. *graminearum* spores. Interestingly, lighter infection and lower fungal growth were also observed for leaves pretreated with indole (Fig 1D and 1F). Such results indicated that indole induced maize resistance against pathogen infection as a priming substance. The priming effect of idole was dose dependent and significant resistance was induced by very low concentration of indole (0.1 mg L^-1^, equivalent to 100 ng g^-1^ *in planta*). The best priming effect was obtained with 50 mg L^-1^ of indole (S2 Fig). We also pretreated detached maize leaves with 50 mg L^-1^ of indole for 24 h, however, no priming effect was observed (S3 Fig). To confirm the priming function of indole in intact leaves, we treated 2-week-old maize seedlings with 50 mg L^-1^ of indole for 2 d and these seedlings were subsequently inoculated with *F*. *graminearum* spores on leaves. These intact leaves also developed milder symptoms and smaller lesions than the control (S4 Fig), indicating the effective priming of indole on intact maize plants.

### Indole induced H_2_O_2_ accumulation in the priming stage

**Fig 2:**
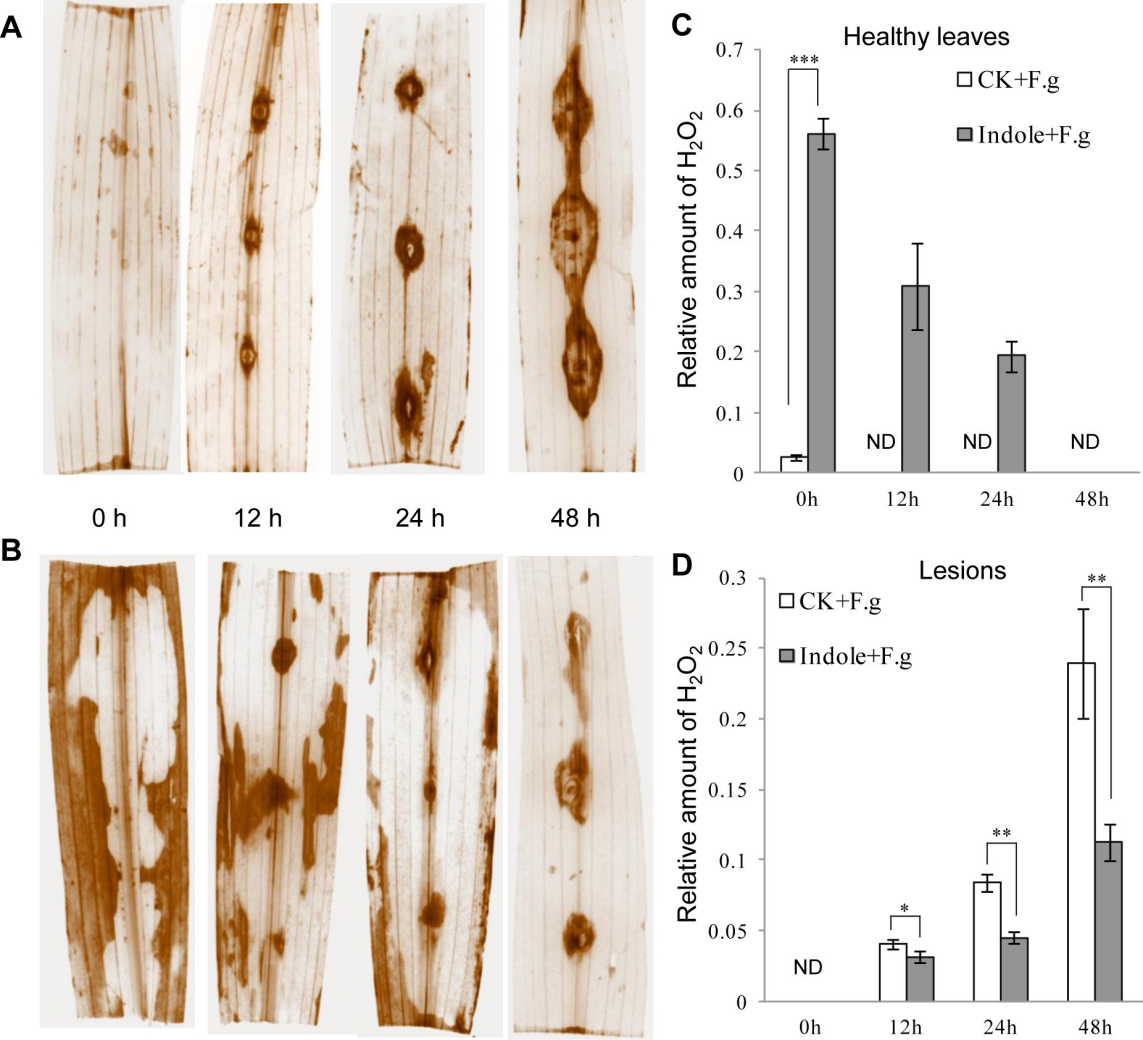
Indole priming resulted in early accumulation and later scavenging of H_2_O_2_. Maize leaves were inoculated with *F*. *graminearum* spores with (**B**, Indole + F.g) or without (**A**, CK+F.g) indole pretreatment and stained with DAB to exhibit H_2_O_2_ accumulation in leaves and infection sites. Spore inoculation time (0, 12, 24 and 48 h) was indicated. Relative amount of H_2_O_2_ at healthy leaf regions (**C**) and lesions (**D**) was calculated based on pixels taken with Photoshop. Asterisks indicate significant difference (Student’s *t*-test, *P<0.05, **P<0.01, ***P<0.001). ND, none detected. Error bars indicate SE (*n* = 3).

H_2_O_2_ is a type of ROS and plays as the secondary messenger in signaling transduction. ROS burst was usually observed in priming [1]. We monitored H_2_O_2_ accumulation by DAB staining in maize leaves after indole pretreatment and significant H_2_O_2_ was observed to generate and distribute randomly at 24 h and 48 h after indole priming but not in the control (Fig 2 and S5 Fig). However, these H_2_O_2_ induced by indole did not cause cell death and leaf lesion and were eliminated later upon spore inoculation (Fig 2C). Continuous observation showed that H_2_O_2_ was induced again by pathogen infection and accumulated specifically at the inoculated sites (Fig 2). In the priming group, less H_2_O_2_ accumulation was observed at the infection spots (Fig 2B and 2D), consistent with smaller infection lesions accordingly (Fig 1D). In contrast, the control group accumulated more H_2_O_2_ and developed larger infection lesions at the infection sites (Fig 2A and 2D).

### H_2_O_2_ induced plant resistance by priming

**Fig 3:**
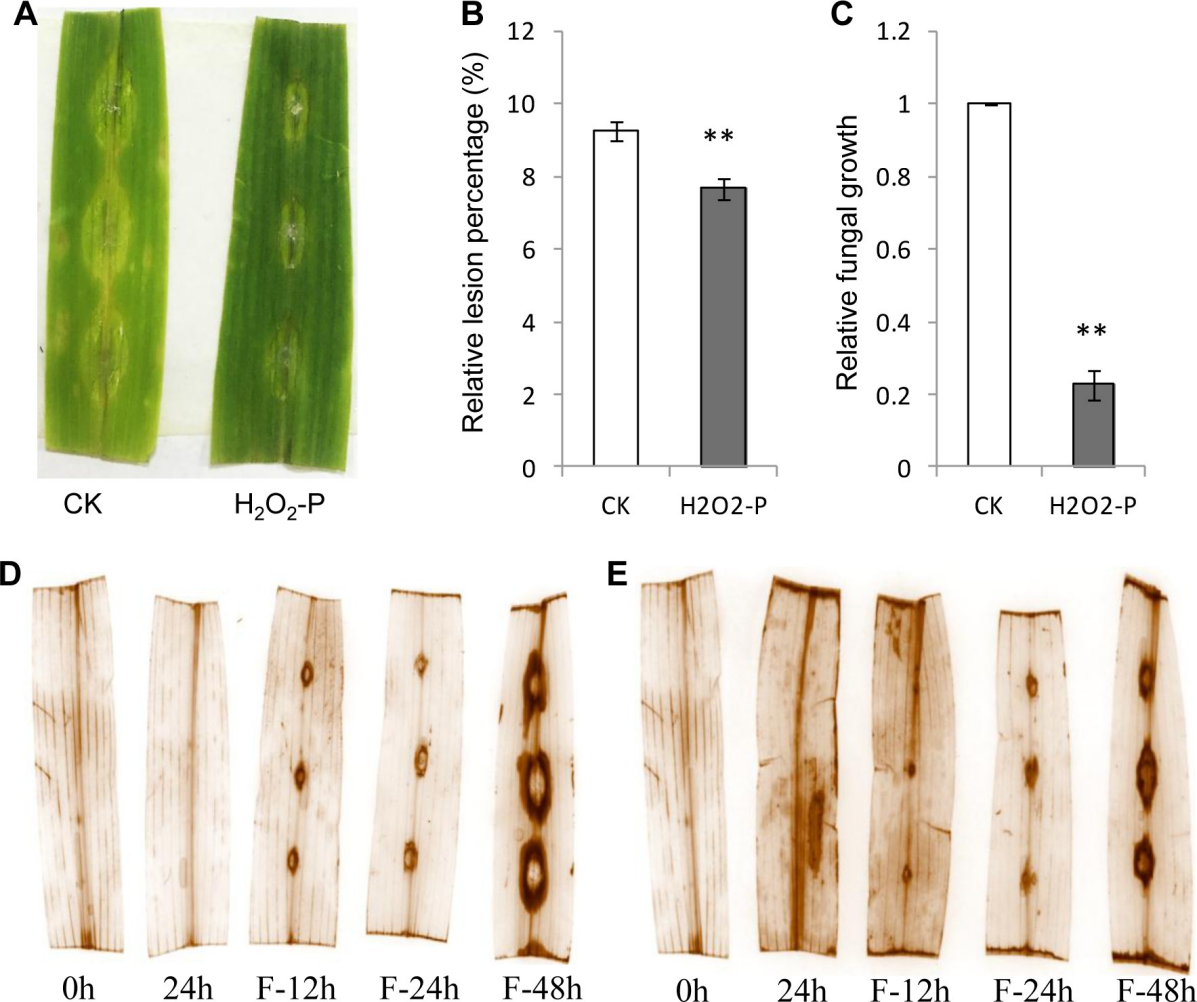
H_2_O_2_ pretreatment induced pathogen resistance. **A**, maize leaves were pretreated with H_2_O_2_ for 24 h and inoculated with *F*. *graminearum* spores for 2 d. Lesions were quantified (**B**) and fungal growth (**C**) was measured by qRT-PCR. H_2_O_2_ accumulation was monitored continuously by DAB staining with (**E**) or without (**D**) H_2_O_2_ pretreatment. Pretreatment (0, 24 h) and subsequent pathogen spore inoculation (F-12 h, F-24 h, F-48 h) were indicated. CK, the control. H_2_O_2_-P, H_2_O_2_ pretreatment. Asterisks indicate significant difference (Student’s *t*-test, **P<0.01). Error bars indicate SE (*n* = 3).

ROS burst is observed in priming process and H_2_O_2_ is identified as the fingerprint molecule for priming [7,9,27]. Indole also induced H_2_O_2_ accumulation here (Fig 2). To explore the role of H_2_O_2_ induced by indole treatment, we used 10 μM H_2_O_2_ to treat detached maize leaves. Preliminary assay indicated that 24 h treatment of H_2_O_2_ was appropriate. Extended treatment (48 h) killed plant cells and these detached leaves exhibited death. After H_2_O_2_ treatment for 24 h, detached leaves were inoculated *F*. *graminearum* spores. Smaller infection lesions were developed than the control (Fig 3A and 3B), as well as lower fungal growth in infected leaves (Fig 3C) and less H_2_O_2_ accumulation at lesions (Fig 3E). We also noticed that H_2_O_2_ treatment induced ROS burst in healthy leaves like indole did (S6 Fig). These results indicated that H_2_O_2_ successfully primed plant defense and might play important role in indole priming.

### MAPK cascade was involved in indole priming

**Fig 4:**
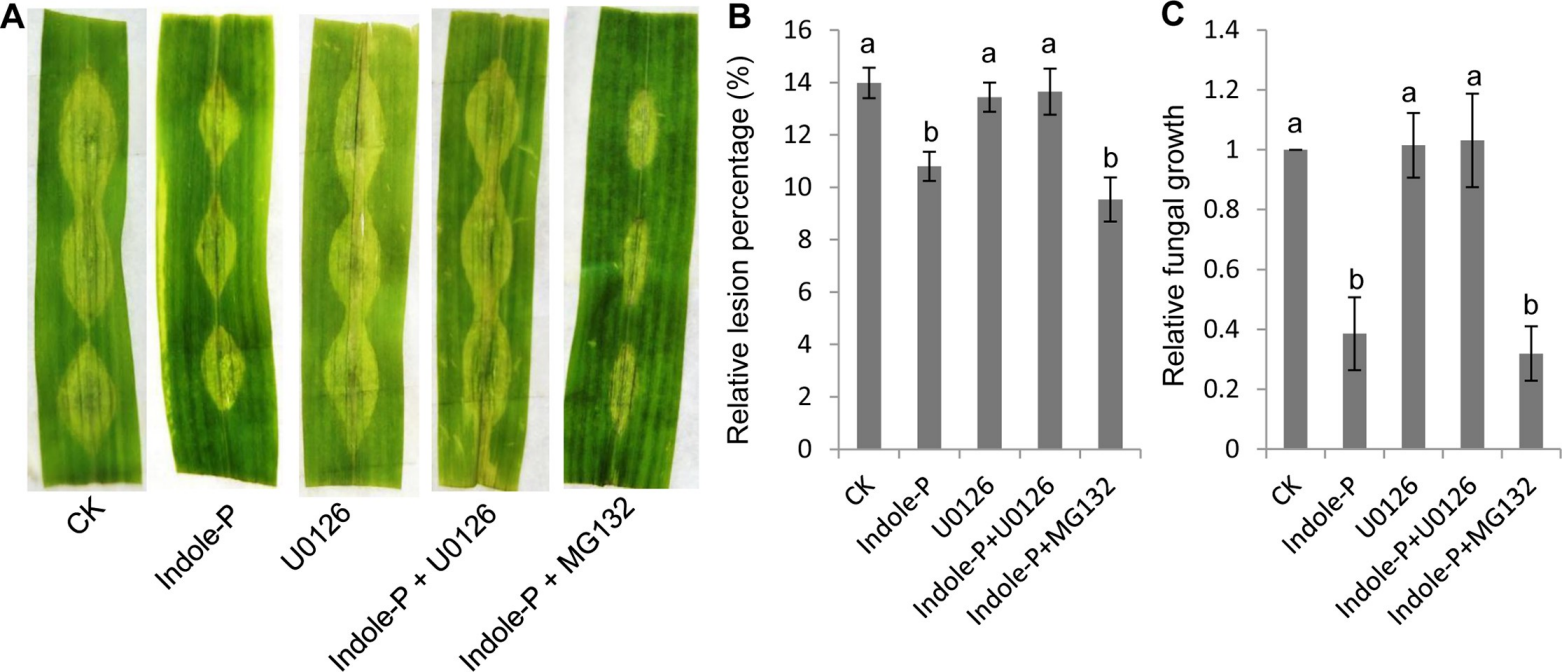
MAPK cascade was essential for indole priming. MAPK cascade inhibitor U0126 or proteasome inhibitor MG132 was applied with indole pretreatment (Indole-P) and priming effect was affected (**A**). Lesions were quantified as relative lesion percentage (**B**). The relative fungal growth was analyzed with qRT-PCR (**C**). None-treated leaves were used as the control (CK). 15 leaves were used for mean value calculation. Different lowercase letters indicate significant difference (Tukey’s HSD test, *P*<0.05, *n* = 4).

MAPK cascade is the important signaling pathway and has been identified to be involved in priming [1,28]. Ubiquitination of suppressor protein followed degradation by proteasome is also the universal mechanism for defensive signaling transduction. Here we used MAPK kinase inhibitor U0126 and proteasome inhibitor MG132 to explore the signaling transduction mechanism in indole priming. U0126 treatment significantly compromised the priming effect of indole and the leaves treated by indole and U0126 developed similar infection to the control (Fig 4). Previous study suggested that inactive MAPK proteins were accumulated in priming stage and converted to be functional kinases through phosphorylation upon pathogen infection [28]. In consistent with previous report, indole priming seemed to involve MAPK cascade in maize leaves against necrotrophic fungi. On the other hand, MG132 treatment did not affect indole priming (Fig 4), indicating that ubiquitination is not essential for indole priming.

### Indole induced early and robust defensive gene expression

**Fig 5:**
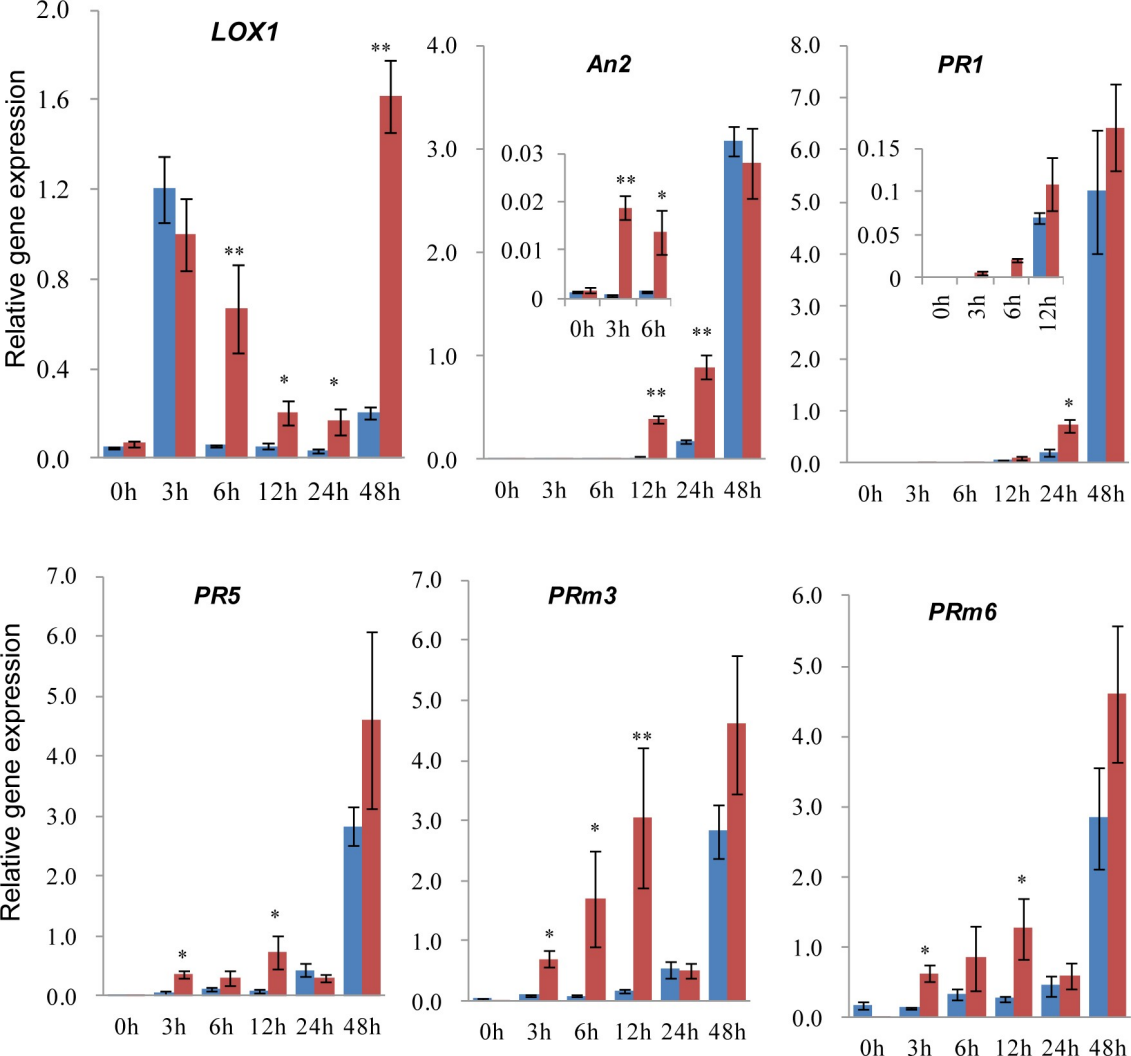
Indole primed defense gene expression upon pathogen infection. qRT-PCR analysis of defense genes in detached maize leaves with pretreatment of indole (priming) for 2 d and subsequent inoculation of *F*. *graminearum* spores. Samples with (red) or without (blue) indole priming were collected at 0, 3, 6, 12, 24 and 48 h after pathogen spore inoculation. Biosynthetic genes *LOX1* for JA, and *An2* for maize phytoalexin were analyzed, as well as PR genes. *Ef1α* was used as the endogenous control. Asterisks indicate significant difference (Student’s *t*-test, *P<0.05, **P<0.01). Error bars indicate SE (*n* = 3).

Priming agents induce earlier and stronger gene expression for defense pathway upon pathogen infection [7,9,27]. To dissect the priming function of indole on gene activation, we analyzed defensive gene expression by qRT-PCR and detected rapid and strong response of plant defense in the primed group (Fig 5). Many defense related genes responded earlier and accumulated more transcripts with indole priming, such as JA synthetic gene *LOX1* (lipoxygenase), maize phytoalexin biosynthetic gene *An2*, *PR* (pathogenesis-related protein) genes including *PR1*, *PR5*, *PRm3* (chitinase), *PRm6* (β-1,3-glucanase) and anti-oxidant enzyme encoding genes. Most of these genes were upregulated at 3 h after spore inoculation in primed samples, which was much earlier than the control (Fig 5). Such inducible gene expression pattern was also reported for other priming substances [6]. In addition, *LOX1* was expressed at 3 h post inoculation both in primed group and the control, which might be resulted from wounding.

**Fig 6:**
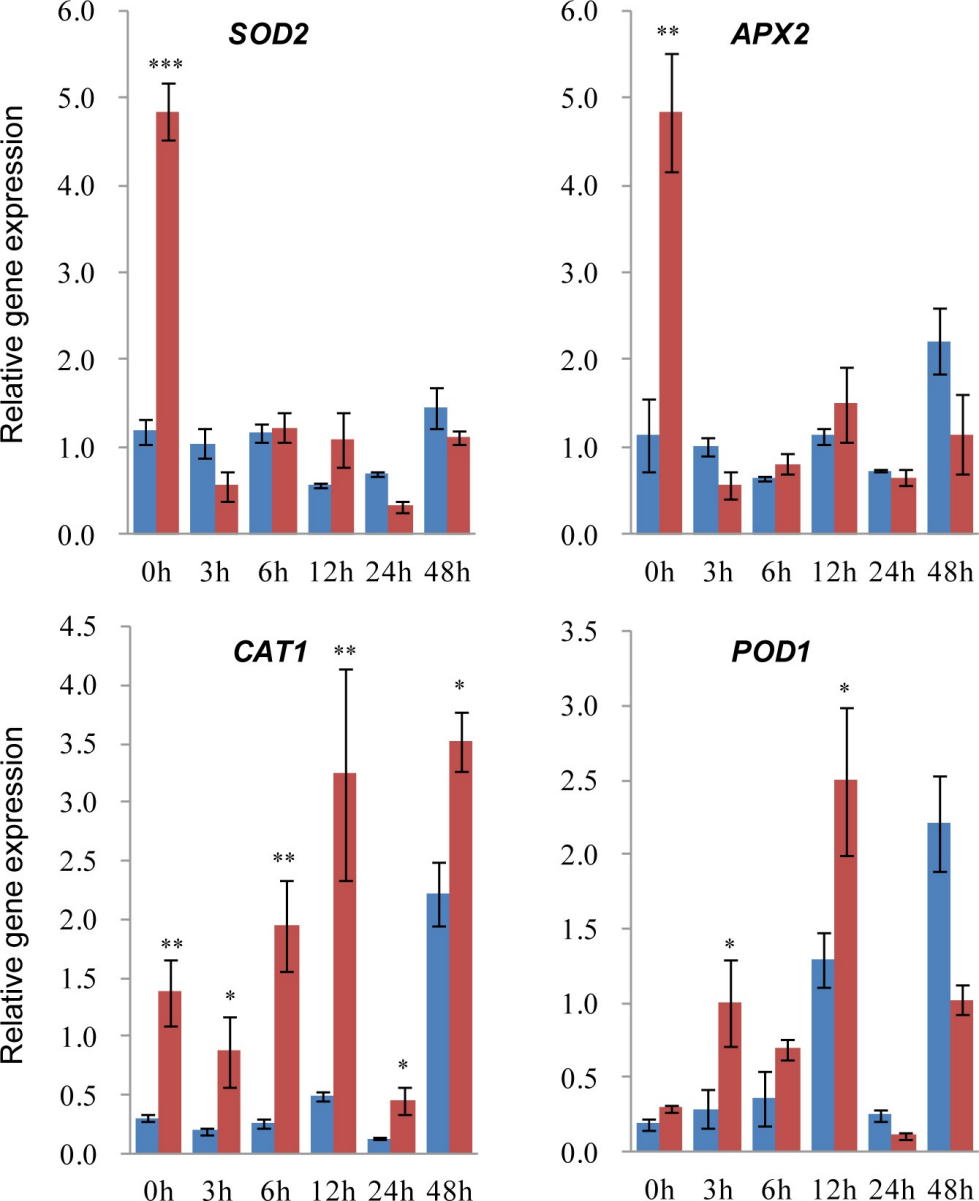
Indole priming induced differential expression of anti-oxidant genes. qRT-PCR analysis of four anti-oxidant enzyme encoded genes in detached maize leaves as described in Fig 5. Primed (red) and non-primed (blue) samples were collected at 0, 3, 6, 12, 24 and 48 h after punch inoculation of *F*. *graminearum* spores. *Ef1α* was used as the endogenous control. Asterisks indicate significant difference (Student’s *t*-test, *P<0.05, **P<0.01, ***P<0.001). Error bars indicate SE (*n* = 3).

In addition, anti-oxidant genes responded in differential patterns, in which *CAT1* (catalase) and *POD1* (peroxidase) were strongly induced at early stage upon pathogen infection in the priming group (Fig 6). However, *SOD* (superoxide dismutase) and *APX* (ascorbate peroxidase) exhibited strong expression only at 0 h after inoculation in the priming group. The different expression pattern of anti-oxidant genes suggests differential regulatory mechanism and roles in Redox equilibrium maintenance. Additionally, high accumulation of SOD and APX in the priming group could be used to erase ROS induced by indole pretreatment as described above (Fig 2).

### Indole priming was a universal mechanism in plant defense

**Fig 7:**
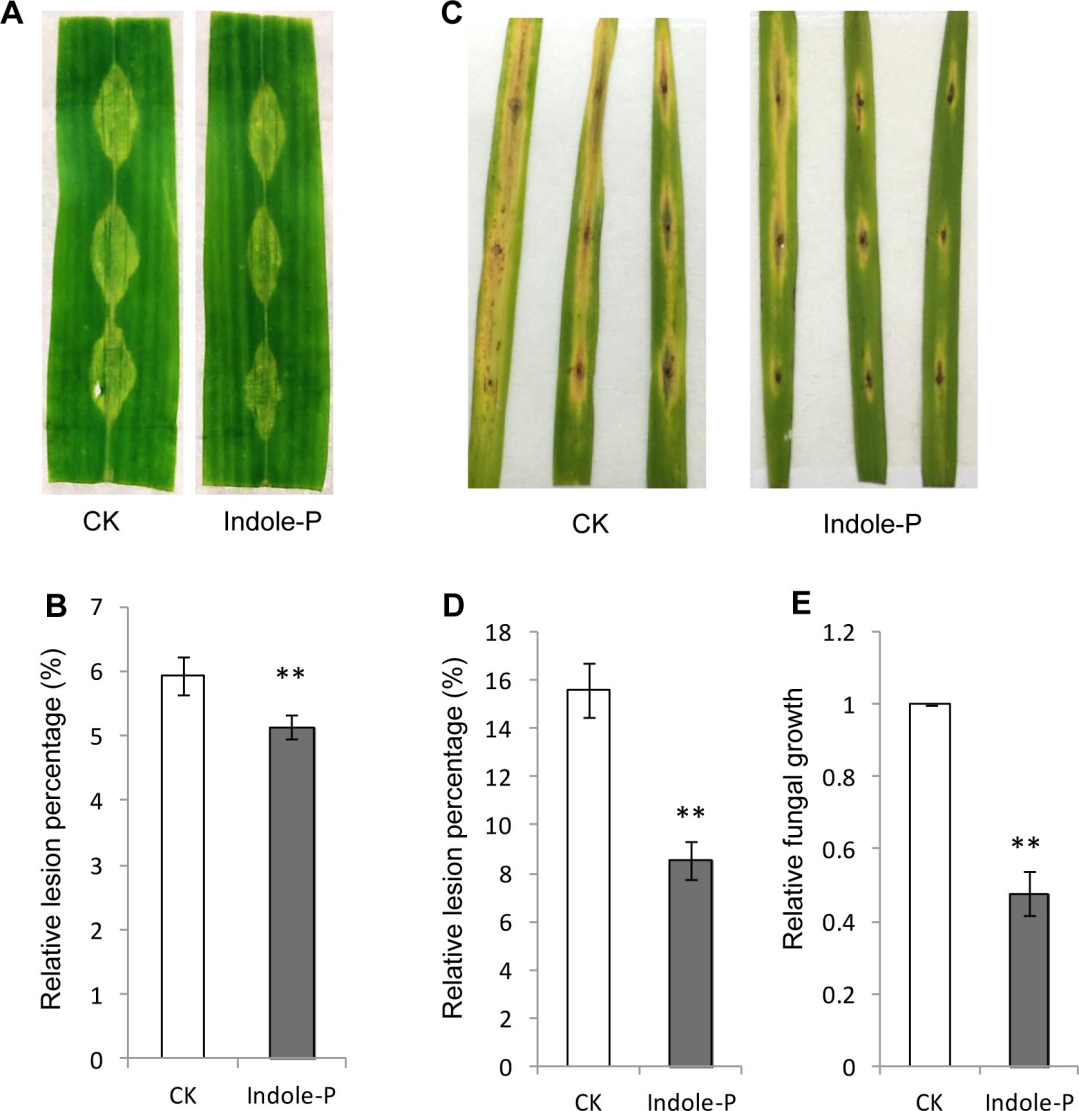
Indole priming induced resistance against *F*. *moniliforme* and *Magnaporthe oryzae*. **A**-**B**, indole pretreatment enhanced resistance against *F*. *moniliforme* in detached maize leaves. **C**-**E**, resistance in detached rice leaves against blast pathogen *M*. *oryzae* was elevated by indole pretreatment. Lesions and fungal growth were quantified (**D**, **E**) as above. ** indicates significant difference (*P<* 0.01, student’s *t*-test). Error bars indicate SE (*n* = 3).

We further tested the priming effect of indole against different pathogens. Indole was also observed to prime resistance against *F*. *moniliforme*, another maize necrotrophic fungus (Fig 7A and 7B). As indole was emitted in rice either, we pretreated detached rice leaves with indole and inoculated with *M*. *oryzae* spores. Unsurprisingly, elevated resistance was monitored in rice pretreated by indole against *M*. *oryzae* (Fig 7C–7E). Hence indole priming might be universal in plants against pathogen infection.

## Discussion

Numerous environmental signals stimulate plants and result in priming establishment, including biotic and abiotic stresses, which enhance defense against pathogen infection, herbivory or abiotic stresses [8]. Many chemical stimuli have been identified as priming agents [6,8,27,29], of which indole was reported to be involved in priming against herbivory [18,22]. Here we identified the priming function of indole in plant defense against necrotrophic fungi. Indole induced defensive systems in maize and resulted in elevated resistance against *F*. *graminearum* and *F*. *moniliforme*, as well as in rice against *M*. *oryzae*. Such priming depended on MAPK cascade and induced H_2_O_2_ accumulation in the priming phase and earlier and stronger defensive gene expression upon pathogen challenge. Concerning wide production of indole in plants [19–21], its priming function and mechanism deserve comprehensive investigation.

The priming mechanism for some priming agents has been characterized [7,8,27]. In the priming phase, many cellular activities are changed to result in accumulation of fingerprint molecules including cytosolic Ca^2+^ and ROS, as well as gene activation. Upon challenged by further stresses after priming, a number of defensive mechanisms are subsequently upregulated with earlier and more robust mode, such as phytoalexins, phytohormones and PR proteins. Indole has been shown to enhance accumulation of phytohormones, terpenes and green leaf volatiles against herbivory [22]. Here indole also activated H_2_O_2_ accumulation in the priming phase and elevated earlier and stronger expression of JA and phytoalexin biosynthetic genes, PR genes and anti-oxidant enzyme encoding genes upon pathogen inoculation (Figs 5 and 6). These cellular and metabolic mechanisms induced by indole are similar to other priming processes.

ROS are the important signals of plant stress response and involved in priming [8,30]. Exogenous elicitors stimulated cytosolic Ca^2+^ concentration change and subsequent signal transduction for ROS generation. ROS either play as the second messengers to activate downstream signals or induce HR. In the mean time, MAPK cascade plays pivotal roles in signal transduction and augmentation via phosphorylation [5,31]. The important member of ROS, H_2_O_2_ has been reported to oxidize cysteine of MAPK in yeast and mammalian to specifically induce downstream transcription factor expression [32], although the mechanism of ROS activating MAPK cascade in plants needs to be elucidated. Here indole priming relied on MAPK cascade and induced H_2_O_2_ burst (Figs 2 and 4). Consistently, indole priming on defense gene expression was compromised after treatment with MAPK inhibitor (S7 Fig). Further analysis identified that H_2_O_2_ also primed plant defense (Fig 3), which might involve MAPK cascade activation. Hence indole treatment induced H_2_O_2_ generation to potentially activate subsequent MAPK cascade, which further phosphorylated downstream protein like transcription factors to activate defensive gene expression. In addition, although indole with high concentration could induce superoxide accumulation to impair cell membrane of bacteria [33], the mechanism of H_2_O_2_ burst caused by indole treatment in plants here remains unclear.

Redox equilibrium is important for keeping normal cell life. Cells rely on ROS scavenging system to abolish ROS and keep redox equilibrium [30]. A massive burst of H_2_O_2_ was observed at lesions of necrotroph infection in maize leaves here, which was correlated with lesion size positively (Figs 1–3). Accordingly, elevated resistance by indole priming resulted in smaller lesions and less H_2_O_2_ accumulation at infection sites. Notably, compared with the control, leaves treated by indole exhibited H_2_O_2_ burst in the priming phase, which did not activate HR to cause cell death and disappeared gradually after pathogen inoculation [30]. In addition, indole priming triggered anti-oxidant gene expression drastically, particularly *CAT* and *POD* for H_2_O_2_ scavenging, upon pathogen infection (Fig 6). These evidences suggest that indole treatment induced earlier and stronger defense via H_2_O_2_ burst to impair fungal growth. The later scavenging of H_2_O_2_ by anti-oxidant enzymes at infection sites also contributed to limit pathogen infection, however, the underlying mechanism of enhanced ROS scavenging remains to be clarified.

Phytohormones play important roles in plant growth/development and environmental adaptation. Priming induces higher phytohormone accumulation and corresponding biosynthetic gene expression [8,27]. JA is involved in plant defense against pest infestation and necrotrophic fungi infection[1]. JA signaling was induced by indole at the priming stage [22]. Here Indole priming also resulted in stronger gene expression of JA biosynthesis than the control after pathogen inoculation (Fig 5), consistent with the defensive role of JA against necrotrophic infection. Furthermore, secondary metabolites like phytoalexins also defend pathogen infection in plants, which have been reported to be induced by priming [8]. Maize phytoalexin biosynthetic gene, *An2* exhibited earlier and stronger expression in primed maize leaves upon pathogen infection, indicating involvement of *An2*-related phytoalexin, kauralexins in elevated resistance by indole priming [34–36].

Plants usually accept challenges simultaneously or continuously from different adverse environmental factors. Cross tolerance is developed in plants to deal with these stresses through recruiting phytohormones and defense mechanisms. ROS have been demonstrated to play important roles in cross tolerance [4]. Indole primed plant defense through H_2_O_2_ induction, suggesting potential involvement in cross tolerance. Insect attack or continuous mechanical wounding resulted in damaged physical barrier of plants, which would be infected by follow-up pathogens. Correspondingly, plants emitted indole upon pest infestation or physical damage to prime defense against not only herbivory but also necrotrophic pathogen infection. Thus, indole might be involved in cross tolerance as a priming agent to induce multiple signaling and plant defense. The following investigation could focus on the molecular mechanism of indole priming and the potential priming ability in response to abiotic stresses.

## Supporting information

S1 Fig No significant toxicity of indole to fungi. (PDF)

S2 Fig Dose dependent priming effect of indole. (PDF)

S3 Fig Indole did not prime defense with 24 h pretreatment on detached maize leaves. (PDF)

S4 Fig Indole primed disease resistance in intact maize seedlings. (PDF)

S5 Fig Indole pretreatment resulted in H_2_O_2_ accumulation in maize leaves. (PDF)

S6 Fig ROS accumulation in maize leaves pretreated with H_2_O_2_. (PDF)

S7 Fig MAPK cascade inhibition compromised indole priming on defense gene expression. (PDF)

S1 Table Primers used in this study. (PDF)
