# Supplementary material for: Indole primes plant defense against necrotrophic fungal pathogen infection
Source: PLoS One. 2018 Nov 16;13(11):e0207607. doi: 10.1371/journal.pone.0207607 (PMC6239302; doi:10.1371/journal.pone.0207607)
Supplement: S1 Fig — (PDF) [file pone.0207607.s001.pdf]

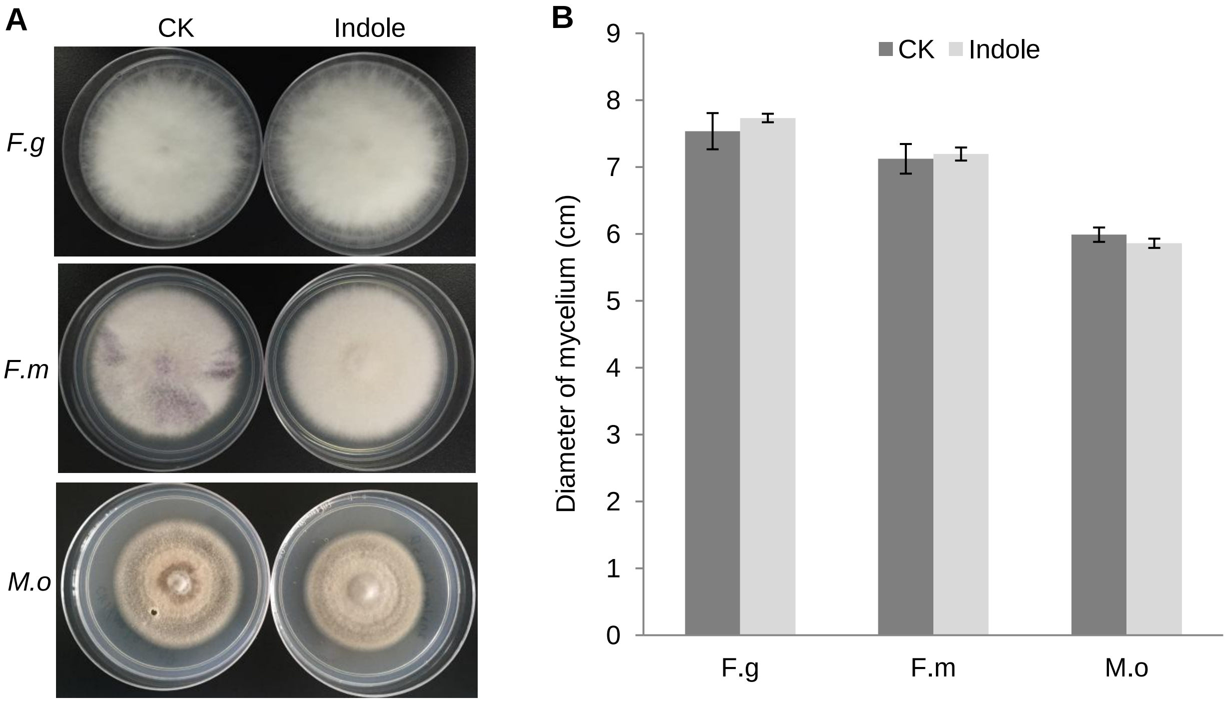

S1 Fig. No significant toxicity of indole to fungi.

Indole was added into PDA medium with the concentration of 50 mg L^{-1}. Three pathogen fungi, *Fusarium graminearum* (*F. g*), *F. moniliforme* (*F. m*) and *Magnaporthe oryzae* (*M.o*) were grown on these plates. Methanol was added as the control (CK). Mycelia (**A**) were observed and their diameters (**B**) were measured and compared. No significant difference was detected with student's *t*-test. Error bars indicate SE (*n*=3).
