# Supplementary material for: Indole primes plant defense against necrotrophic fungal pathogen infection
Source: PLoS One. 2018 Nov 16;13(11):e0207607. doi: 10.1371/journal.pone.0207607 (PMC6239302; doi:10.1371/journal.pone.0207607)
Supplement: S2 Fig — (PDF) [file pone.0207607.s002.pdf]

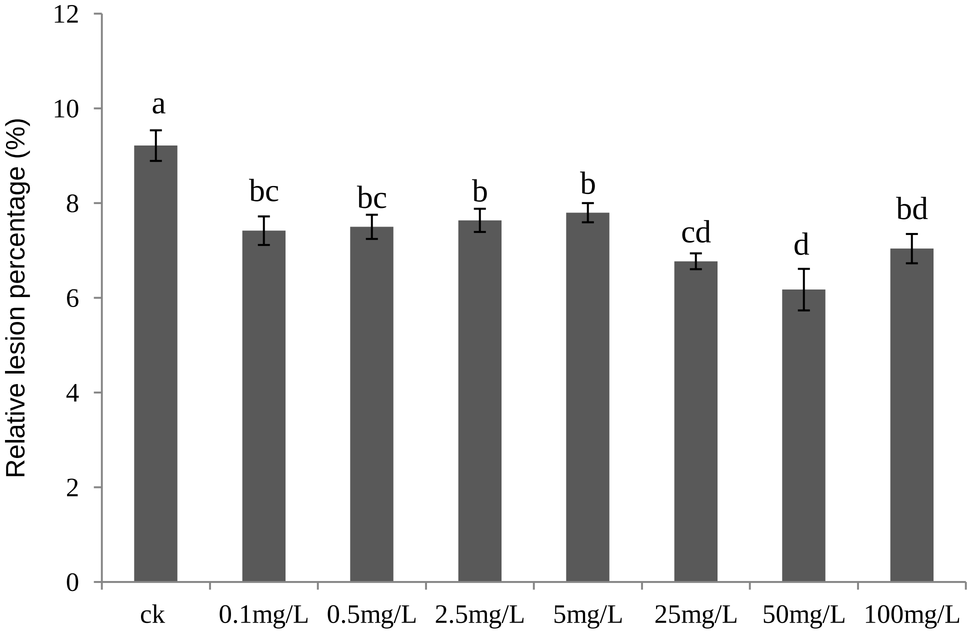

S2 Fig. Dose dependent priming effect of indole.
Detached maize leaves were pretreated with indole at different concentrations for 2 d and subsequently inoculated with *F. graminearum* spores for 3 d and relative lesion percentage was calculated. Different lowercase letters indicate significant difference (Tukey's HSD test, $P<0.05$, $n=4$).
