# Supplementary material for: Indole primes plant defense against necrotrophic fungal pathogen infection
Source: PLoS One. 2018 Nov 16;13(11):e0207607. doi: 10.1371/journal.pone.0207607 (PMC6239302; doi:10.1371/journal.pone.0207607)
Supplement: S3 Fig — (PDF) [file pone.0207607.s003.pdf]

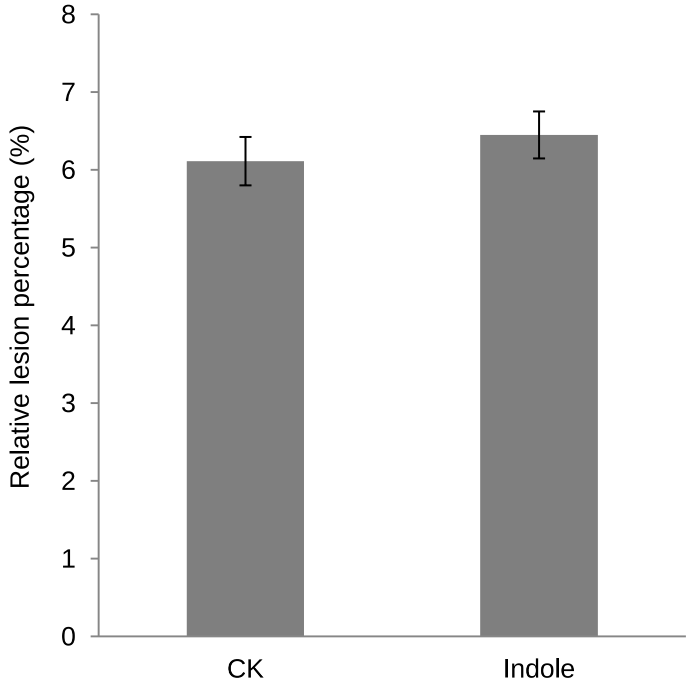

S3 Fig. Indole did not prime defense with 24 h pretreatment on detached maize leaves. Indole (50 mg L^{-1})was used to pretreat detached maize leaves for 24 h and *Fusarium graminearum* spores were inoculated. None-treated leaves were used as the control (CK). Relative lesion percentage was calculated and no significant difference was detected with student's *t*-test. Error bars indicate SE (*n*=3).
