# Supplementary material for: Indole primes plant defense against necrotrophic fungal pathogen infection
Source: PLoS One. 2018 Nov 16;13(11):e0207607. doi: 10.1371/journal.pone.0207607 (PMC6239302; doi:10.1371/journal.pone.0207607)
Supplement: S4 Fig — (PDF) [file pone.0207607.s004.pdf]

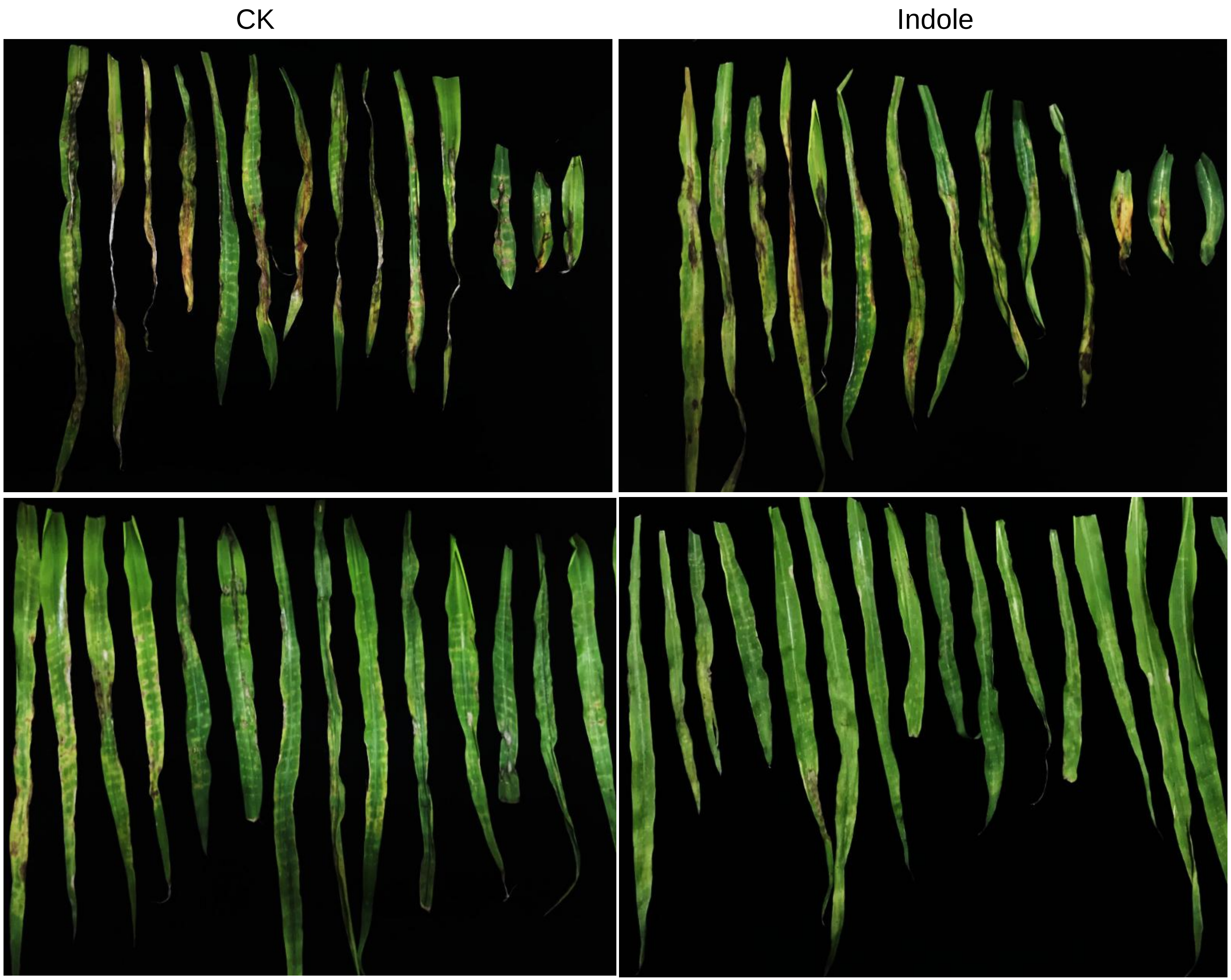

S4 Fig. Indole primed disease resistance in intact maize seedlings.
Two-week-old maize seedlings were pretreated with indole and inoculated with *F. graminearum* spores for 3 d and leaves were cut for photographing.
