# Supplementary material for: Indole primes plant defense against necrotrophic fungal pathogen infection
Source: PLoS One. 2018 Nov 16;13(11):e0207607. doi: 10.1371/journal.pone.0207607 (PMC6239302; doi:10.1371/journal.pone.0207607)
Supplement: S5 Fig — (PDF) [file pone.0207607.s005.pdf]

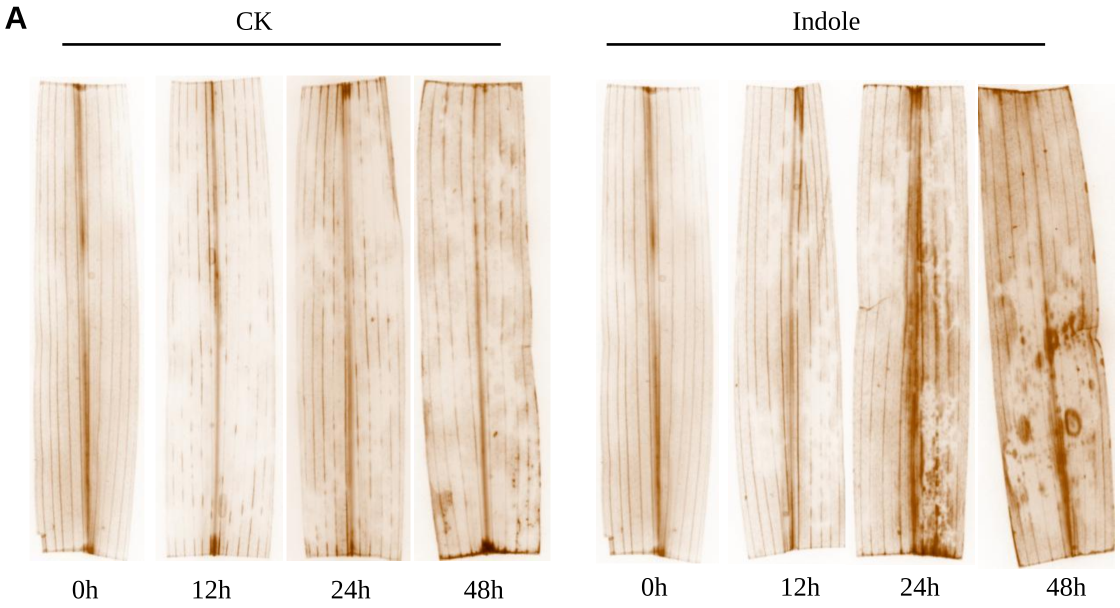

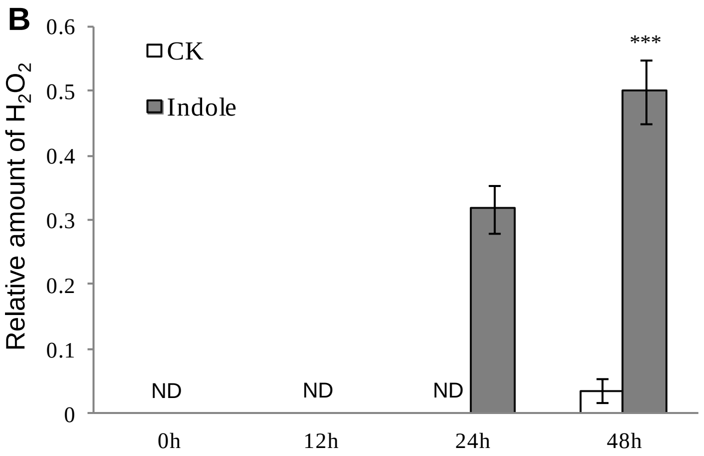

S5 Fig. Indole pretreatment resulted in H_2O_2 accumulation in maize leaves.
A, H_2O_2 accumulation by DAB staining in maize detached leaves with (Indole) or without (CK) indole pretreatment for 0, 12, 24 and 48 h. **B**, quantification of H_2O_2. Asterisks indicate significant difference (Student's *t*-test, ***$P<0.001$). ND, none detected. Error bars indicate SE (n=3).
