# Supplementary material for: Indole primes plant defense against necrotrophic fungal pathogen infection
Source: PLoS One. 2018 Nov 16;13(11):e0207607. doi: 10.1371/journal.pone.0207607 (PMC6239302; doi:10.1371/journal.pone.0207607)
Supplement: S6 Fig — (PDF) [file pone.0207607.s006.pdf]

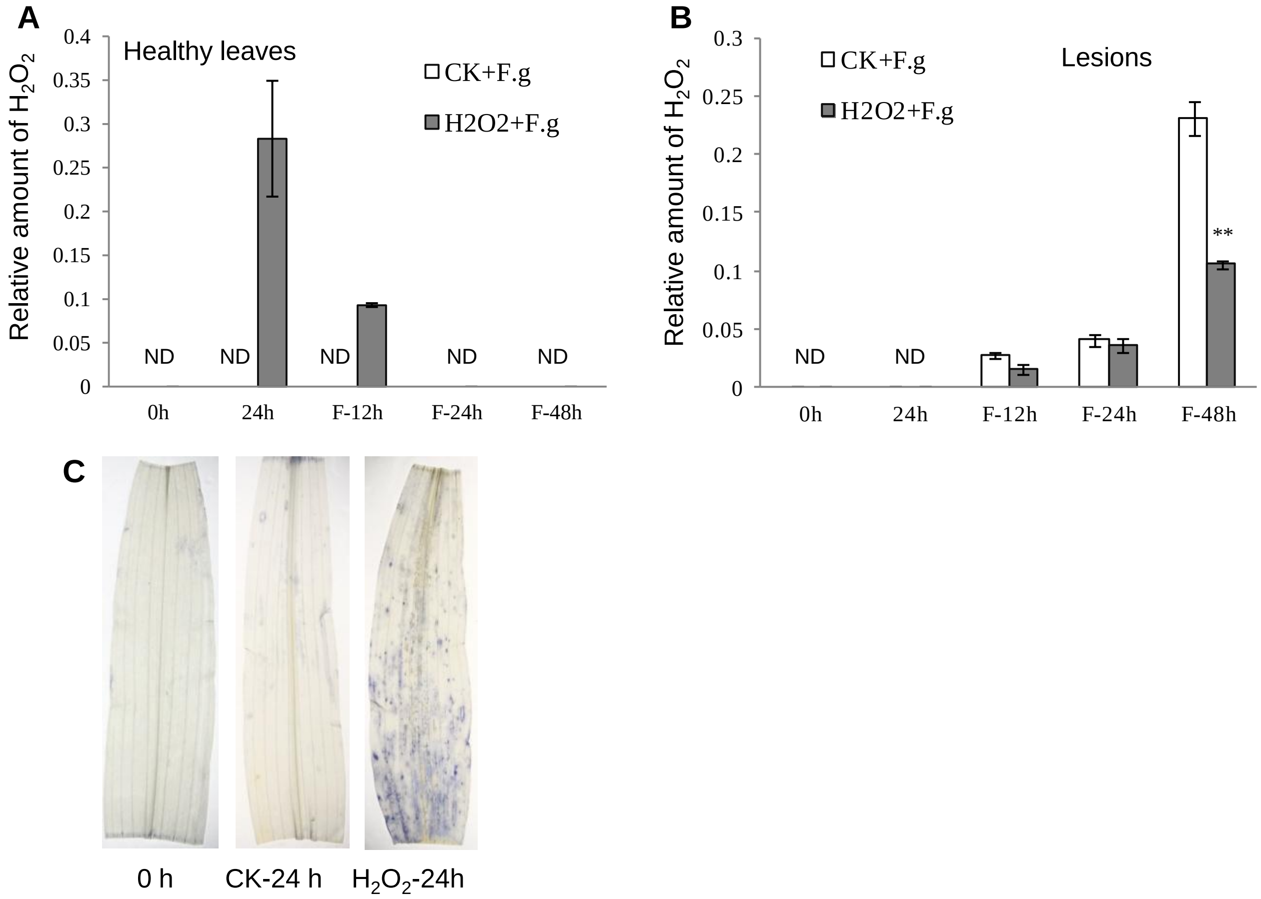

S6 Fig. ROS accumulation in maize leaves pretreated with H_2O_2.

H_2O_2 accumulation by DAB staining in maize detached leaves with or without (CK) H_2O_2 pretreatment for 0, 24 h and subsequently inoculated with pathogen spores (F.g) for 12, 24 and 48 h (F-12h, 24h, 48h) as shown in Fig.6. Relative amount of H_2O_2 at healthy leaf regions (**A**) and lesions (**B**) was calculated based on pixels taken with Photoshop. Asterisks indicate significant difference (Student's *t*-test, $^{**}P<0.01$). ND, none detected. Error bars indicate SE ($n=3$). **C**, Superoxide radical (O_2^-) was stained with NBT after H_2O_2 pretreatment for 24 h.
