# Supplementary material for: Indole primes plant defense against necrotrophic fungal pathogen infection
Source: PLoS One. 2018 Nov 16;13(11):e0207607. doi: 10.1371/journal.pone.0207607 (PMC6239302; doi:10.1371/journal.pone.0207607)
Supplement: S7 Fig — (PDF) [file pone.0207607.s007.pdf]

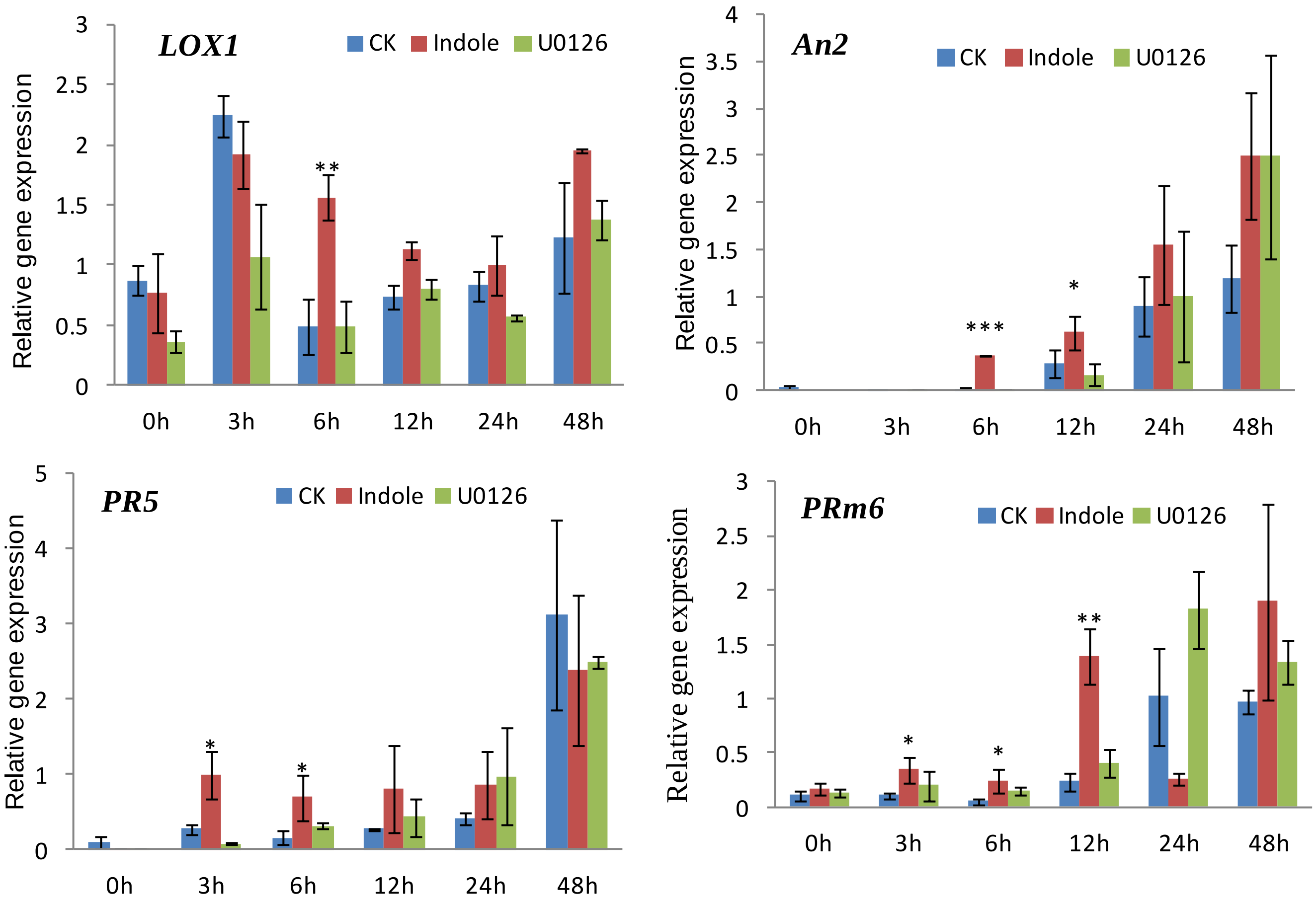

S7 Fig. MAPK cascade inhibition compromised indole priming on defense gene expression. qRT-PCR analysis of defense gene expression in detached maize leaves inoculated with *F. graminearum* spores after indole priming (indole) or MAPK inhibitor (U0126) treatment plus indole priming. CK was the control without indole or MAPK inhibitor treatment. The pathogen spore inoculation time was indicated (0, 3, 6, 12, 24 and 48 h). Asterisks indicate significant difference (Student's *t*-test, **P*<0.05 ,***P*<0.01, ,****P*<0.001). Error bars indicate SE (*n*=3).
