# Supplementary material for: Indole primes plant defense against necrotrophic fungal pathogen infection
Source: PLoS One. 2018 Nov 16;13(11):e0207607. doi: 10.1371/journal.pone.0207607 (PMC6239302; doi:10.1371/journal.pone.0207607)
Supplement: S1 Table — (PDF) [file pone.0207607.s008.pdf]

S1 Table Primers used in this study.

Gene	Forward Primers(5'->3')	Reverse Primers (5'->3')
ZmPR1	GAACTCGCCGCAGGACTAC	GAGCCCCAGAAGAGGTTCTC
ZmPR5	GTCATCGATGGCTACAACCT	GGGCAGAAGGTGACTTGGTA
ZmPRm3	GGCTCTACGCCTACGTCAAC	GATGGAGAGGAGCACCTTGA
ZmPRm6	GCGCAGACCTACAACCAGA	GGAGAAATTGATGGGGTACG
ZmAn2	GATGATGAGCCATGTCGATG	GAAAGGTCTGCCTTGTCTCG
ZmTPS6	CGGTGATCAATGAGCCACTA	TGACAACATGTGCCAACTCC
ZmLOX1	AGCATCTCCATCTGATCCATCC	CGTGCCTCTTGTTGCATTGA
ZmPAL3	TCCTGTCCGCCGTCTTCTGC	CGGGTTGTCGTTCACGGAGTT
ZmSOD2	CGGGAGAAGATGGTGTTGTT	ATTTCCGGTGCTCTTGCTAA
ZmPOD1	GCACAAGGTCCTGTTCGTCT	TTTCCCTGATCTCTCCCTCA
ZmCAT1	GTGAATGCACCAAAATGTGC	TGATGCACTTCTCACGACAG
ZmAPX2	CCCATCCTATCCTACGCTGA	AGAAAACCTGCCTGAGGTGA
ZmEf1α	TGGTGTCATCAAGCCTGGTA	AACATTGTCACCCGGAAGAG
MoPot2	ACGACCCGTCTTTACTTATTTGG	AAGTAGCGTTGGTTTTGTTGGAT
OsUbq	TTCTGGTCCTTCCACTTTCAG	ACGATTGATTTAACCAGTCCATGA
ZmEf1α-DNA	TCTCTGGGTTTGAGGGTGAC	GGCCCTTGTACCAGTCAAGGT
FgEf1α	CCATTCCCTGGGCGCT	CCTATTGACAGGTGGTTAGTGACTGG
